# Society Meetings

**Published:** 1914-08

**Authors:** 


### SOCIETY MEETINGS

Brief reports and announcements of meetings of societies of Western New York, and those of general scope, are requested from Secretaries. Copy should be on hand the fifteenth of the month. Full transactions will be published at cost of composition.

### Report of the 39th Annual Meeting of the Alumni Association Medical Department, University of Buffalo.

The 39th annual meeting of this association took place Tuesday to Friday, June 2 to 5, 1914. All of the sessions with the exception of the annual Alumni dinner and the Frat reunions were held in the Alumni Hall which was decorated with U. of B. banners. In point of attendance, timeliness and importance of topics discussed at the scientific sessions, in interest and enthusiasm displayed by the Alumni, the meeting was the most successful since the new method of holding same has been in vogue.

SCIENTIFIC PROGRAM

The scientific program consisted of papers and demonstrations given on Wednesday and Thursday. The contributions were all of a high scientific character, the subjects discussed were extremely practical and the entire program was listened to with much interest by the Alumni present.

**Wednesday afternoon, June 3rd.**

Our distinguished fellow Alumnus Dr. Edward N .Brush, '74 who has devoted his entire life to the study of Neurology and Psychiatry and is superintendent of and physician-in-chief to the Sheppard and Enoch Pratt Hospital at Towson, Md., read a paper on **The Trend of Modern Psychiatry.** After briefly reviewing the history of Insanity and the methods of treatment, he discussed very fully the recent advances in modern methods of study as well as therapy. Due consideration was given to syphilis as an etiological factor and the present means in diagnostic methods made possible by the study of the blood and cerebro-spinal fluid. The paper was discussed by Drs. Herman G. Matzinger, '84, James W. Putnam, '82, and others.

Dr. Frederick H. Pratt, Prof. of Physiology who has been in our school for two years and has proven his ability as a teacher and investigator, read a paper on **The Principles Underlying the Clinical Determination of Blood Pressure.** He reviewed the origin and significance of arterial tension and emphasized the importance of finding both systolic and diastolic pressure in clinical work. Methods of determining these were discussed, special reference being made to the ways of reading diastolic pressure, particularly by the auscultatory method. The recent interesting experiments by MacWilliam and Melvin with fresh arterial tissue in connection with an artificial medium were outlined and the bearing of their results on current theories as to the behavior of the compressed artery under the armlet of the syphgmomanometer were explained with the aid of diagrams. According to these authors, the indicator for reading diastolic pressure by the auscultatory method is proved to be the beginning of the last phase of pulse sounds. The sounds are not produced in the distal artery, but wholly beneath the armlet. The discussion of this valuable paper was opened by Dr. DeLancey Rochester, '84.

Dr. A. A. Thibaudeau, Associate Professor of Bacteriology and Hygiene, in his paper gave an unusually interesting and lucid discussion of **The Practical Value of Serum Reaction in Diagnosis.** He divided the important serum reactions into four groups, viz.: 1—Agglutination reactions such as used in the diagnosis of Typhoid, Paratyphoid, Cholera, etc. 2—Reactions to determine opsonic indices, (seldom used in this country). 3—Complement fixation reactions, such as the Wasserman reaction for Lues and the complement fixation test for the gonorrhoeal antibody. 4—Reactions to determine the presence or absence of specific proteolytic ferments in the serum **(Abderhalden reactions).** In the third and fourth groups special emphasis was laid on the great need for a standardization of the methods in the technic of these tests. The value of the Abderhalden reactions depends entirely on an improved technique before they can be made absolutely positive. Dr. Charles A. Bentz, '02, opened the discussion.

---

### Thursday, June 4th.

Thursday was essentially our big day. The first paper, **X-Ray Diagnosis of Surgical Lesions of the Stomach and Duodenum,** was given by Dr. George Emerson Brewer, '84, and Dr. Lewis Gregory Cole, both of New York City. Mention should be made of the prominence which our fellow Alumnus, Dr. Brewer, has attained in surgery both in this country and abroad. Dr. Brewer has recently been appointed Professor of Surgery in the College of Physicians and Surgeons, Columbia University and Surgeon-in-chief to the Presbyterian Hospital, both of New York City. He was president of the American Surgical Association recently held in New York City, and will preside at the meeting of the North American Congress of Surgeons in London England this summer. The paper was the result of considerable clinical study made jointly by both collaborators, Dr. Brewer investigating the clinical and surgical phases whilst Dr. Cole studied the roentgenological aspect of the cases. As each case was related by Dr. Brewer, the lantern slide was thrown on the screen and the features pointed out by Dr. Cole. After the completion of the set paper, Dr. Cole discussed the radiographic aspect and then presented a series of moving pictures demonstrating the actual shape and size of the stomach in vito and incidentally the peristaltic wave action. The method of illustration of both paper and cinematographs showed the masterly skill of Dr. Cole both as a radiographer and interpreter. Dr. Allen A. Jones, '89, opened the discussion which was participated in by a large number of members present after which Drs. Brewer and Cole closed the discussion.

After the luncheon tendered by the Faculty in the College Library, Dr. Herman M. Biggs, Health Commissioner of the State of New York, who has devoted so much time, attention and energy to the study of Hygiene and Sanitation explained **The New Public Health Law of New York State and Its Administration.** This law was largely the result of Commissioner Biggs activities and it was intensely interesting and exceedingly valuable to our members many of whom are health officers or in the public health service to have explained to them the many intricacies of this law. Surely those who heard the doctor fully appreciate the significance and importance of prevention in modern medicine. The discussion was opened by Dr. Edward Clark, '80, for many years in public health service and now District Health Officer in this vicinity under Commissioner Biggs. Health Commissioner Fronczak, '97, of Buffalo, and others participated in the discussion.

Dr. John A. Fordyce, Professor of Dermatology and Syphilology, College of Physicians and Surgeons, New York City, than whom no one is better conversant with the ravages of Lues on mankind, gave a most excellent dissertation on **The Treatment of Syphilis of the Nervous System.** After relating the etiology and symptomatology referable to the nervous system, Prof. Fordyce spoke on the importance of the Wasserman reaction in the blood and spinal fluid, the cell count and globulin reaction in the latter, likewise the recently developed collodial gold test of Lange in relation to the diagnosis and their importance both with and apart from the clinical findings. The more modern methods of treatment, viz.: Salvarson and Neo-Salvarsan intramuscularly and intravenously administered as well as the intra-spinous method of injection of autosalvarsanized serum and the effects of same in nervous and mental syphilis were fully explained. Drs. James W. Putnam, '82, and Henry C. Buswell, '88, discussed the paper,

### Demonstrations in Physiological Laboratory

Professor Pratt and his assistants gave demonstrations on Wednesday and Thursday noon to groups illustrative of **The Action of Heart Valves** with application to teaching. By means of Gad's method, the valves of the left side in an ox heart were rendered visible by inserting "windows" in auricle and aorta and illuminating the ventricular cavity by an incandescent light. Ventricular pressure being artificially raised, water was pumped thru a circuit of glass and rubber tubing permitting the valves to functionate in a manner approximating the normal. Methods of employing this in teaching were described and it was shown how typical pressure curves could be obtained with a manometer, and the periods of valve-action compared with them. Incompetency of valves was also artificially produced by drawing back one or more of the valve-flaps with wire hooks,—the results being clearly visible and readily comprehended by the observer.

### THE HARRINGTON LECTURES

The Harrington Lectures established by the late Dr. D. W. Harrington, '71, in a fund entrusted to the Faculty are given from time to time by men of prominence and distinction in the medical profession of America and Europe. Excluding this year's series there have been three sets of lectures; first series in 1908 by Dr. Simon J. Meltzer of New York City on "The Physiology of Lymph and Pathology of Edema." The second series in 1910 by Professor J. Clarence Webster of the Rush Medical College of Chicago on the History of Obstetrics and Gynecology and the third series in 1912 by Dr. Ludwig Hektoen, Professor of Pathology, Rush Medical College, Chicago, on Immunity in its Relation to Surgery. For the present or Fourth series the Faculty selected Professor Dr. Ludwig Pick, the noted Pathologist of the University of Berlin, Germany. Professor Pick, under the caption, **Recent Advances in Pathological Anatomy,** delivered three lectures on Tuesday, Wednesday and Thursday, June 2, 3, and 4, the respective titles of which were **Neuro-Blastomata, Pseudo-hermaphroditism** and **True Hermaphroditism.** The lectures were delivered in English, in a diction that was faultless and a manner that was both entertaining and effective, so much so that the attention of the audience was sustained throughout. The lectures were illustrated with special lantern slides made from drawings of actual specimens, from colored photo-micrographs, and Lumiere plates, the particular features being the sharp definition, the clear coloring and the stereoscopic effect. Those who were so fortunate as to hear this scholar and scientist will surely carry with them an appreciation of Germany's contribution to medical science.

It is the intention of the faculty to publish these lectures in the near future as soon as the corrected copy is forwarded to this country by Professor Pick.

### ANNUAL BUSINESS MEETING

The annual business meeting was held on Wednesday morning, June 3d, Dr. Grover W. Wende, presiding. Dr. L. Kauffman, chairman, in submitting the annual report for the Executive Committee related the activities during the past year in the management of the affairs of the association. In compliance with resolution adopted last year two branch Alumni Associations were founded in conjunction with the other departments, viz.: the **Interstate Alumni Association** comprising graduates of all departments of the U. of B. residing in Southern New York and Northern Pennsylvania and the **Rochester District Alumni Association** consisting of all U. of B. graduates residing in Rochester and vicinity.

With reference to incorporating our Association careful research by our secretary, Dr. Julius Richter, revealed the fact that this association was duly incorporated in 1875 as per records in County Clerk's office. This made it unnecessary for further action. Two separate constitutions and by-laws with distinctive features were drafted by the committee to present at this meeting for adoption, but on Thursday, May 28, the old Constitution and By-laws were unearthed in the safe of the Faculty. The committee with only five days before the meeting proceeded to make such changes as were deemed necessary to have this constitution and by-laws conform to the present requirements of the Association. The amendments to the constitution of necessity were laid on the table for adoption at our next annual meeting in 1915, but the by-laws were changed so that our Alumni will have the privilege of voting for the amended constitution next year. The following resolutions were recommended by the Executive Committee and unanimously adopted:

1—That branch Alumni Associations of all the departments of the University of Buffalo with local organization be formed where in the judgment of your Executive Committee in conjunction with the officers of the other departmental Alumni, same be deemed advisable.

2—That the formation of a Federated Alumni Association of all departments of the U. of B. be perfected if possible the coming year.

3—That in connection with the University Day celebration an annual dinner of the Federated Alumni Association of all the departments of the U. of B. be held each year.

4—That the Faculty be requested to publish this year a catalogue of the Alumni of our department.

With the co-operation of our Alumni we will organize a branch Association in Western New York and Western Pennsylvania and also in Central and Northern New York with Syracuse, Utica and Watertown as a nucleus. A joint Alumni dinner on next University Day of all the departments will surely tend to develop the spirit of enthusiasm and loyalty in all U. of B. graduates residing in Buffalo and vicinity and incidentally show the citizens of Buffalo how great a factor for education our Alma Mater really is.

It was unanimously adopted that life membership in the Association be henceforth abolished, as the dues of the Association are inadequate to pay the expenses of carrying out the manifold purposes desired. We trust that from now on every Alumnus will be imbued with the spirit of loyalty and send his dues ($1.00 per annum) when requested. Officers for the ensuing year were elected as follows:

President, George F. Cott, '84, Buffalo, N. Y.
First Vice-president, Robert T. French, '88, Rochester, N. Y.
Second Vice-president, William House, '95, Portland, Ore.
Third Vice-president, Abram T. Kerr, '97, Ithaca, N. Y.
Fourth Vice-president, Burt C. Johnson, '90, Buffalo, N. Y.
Fifth Vice-president, Mary B. Moody, '76, Los Angeles, Cal.
Secretary, Julius Richter, '04, Buffalo, N. Y.
Trustee for Five Years, Grover W. Wende, '89, Buffalo, N. Y.
Executive Committee—Lesser Kauffman, Chairman, Edith R. Hatch, '06; Bernard F. Schreiner, '09, all of Buffalo.

Next year our officers will be elected by ballot with two tickets in the field. Opportunity will be afforded every Alumnus in good standing, i. e. who has paid dues to date, to cast his or her ballot by mail if unable to attend the meeting in person.

### MEMORIAL HOUR

Owing to the death, this year, of our dearly beloved teacher and friend, Professor Roswell Park, and Dr. Frederick C. Busch, a special Memorial Hour was held on Thursday, June 4, at ten a. m. President Wende requested Dr. George Emerson Brewer the First Vice-president to preside. In his introductory remarks Dr. Brewer expressed our great loss in the death of Professor Park as well as the void left in American and International Surgery.

Secretary Richter read the **Necrology List** for the past year there being 24 deaths in our ranks.

### Honorary Member

ROSWELL PARK, A. M., M. D., LL. D., Professor of the Principles and Practice of Surgery and Clinical Surgery in the University of Buffalo, beloved and revered teacher of our Alumni, died at his home in Buffalo, N. Y., of heart disease, February 15, 1914, age 61.

**Active Members**

Class of

1851 WALTER SCOTT HICKS. Died in Bristol, N. Y., March 16, 1914. Age, 86.

1860 CHARLES W. CARRIER. Died in Troy, Pa., March 24, 1914. Age, 73.

1863 DASIOM A. FARRINGTON. Died in Holland, N. Y., December 30, 1914. Age, 76.

1864 IRA L. JONES. Died in Minetto, N. Y., July 30, 1913. Age, 81.

1866 LEWIS BLANCHARD. Died in Edgewood, Iowa, August 5, 1913.

1869 JOHN W. DEE. Died in Niagara Falls, Ont., December 2, 1913. Age, 80.

1869 IRA D. HOPKINS. Died in Utica, N. Y., February 23, 1913. Age, 81.

1872 ISAAC E. BENNETT. Died in Plano, Ill., February 19, 1914. Age, 66.

1875 JOHN A. McDONELL. Died in Chicago, Ill., September 19, 1913. Age, 66.

1877 GEORGE S. WYCKOFF. Died in Pittsburgh, Pa., February 10, 1914. Age 63.

1879 LUCIEN E. ELLIS. Died in Detroit, Mich., July 12, 1913. Age, 63.

1880 KAY A. SWEET. Died in Bradford, Pa., April 1, 1914. Age, 61.

1881 WILLIAM H. PARKER. Died in Santa Monica, Cal., March 20, 1914. Age, 59.

1881 JOHN B. HOYER. Died in Middleport, N. Y., April 13, 1914.

1884 VINCENT G. HAMILL. Died in New York City, March 25, 1914. Age, 56.

1887 J. W. E. K. DAVIS. Died in Omaha, Neb., December 4, 1913. Age, 64.

1890 HARRIE B. HOWELL. Died in Rochester, N. Y., February 6, 1914. Age, 50.

1892 CHARLES E. BOULT. Died in Honeoye Falls, N. Y., November 7, 1913. Age, 54.

1895 EDWIN C. REAMES. Died in Canastota, N. Y., September 25, 1913. Age, 43.

1897 FREDERICK C. BUSCH. Died in Buffalo, N. Y., January 3, 1914. Age, 40.

1899 BERNARD F. DENNIS. Died in Oil City, Pa., August 1, 1913. Age, 36.

1900 ALEXANDER M. TROUP. Died in Holland, N. Y., August 24, 1913. Age, 35.

1901 WILLIAM R. PATTERSON. Died in Lloydell, Pa., September 6, 1913. Age, 39.

1906 EDWARD ENOS HOPKINS. Died in Honeoye Falls. N. Y., October 24 ,1913.

### ROSWELL PARK

Dr. Edgar R. McGuire, '01, associated with Dr. Park in teaching and practice for the past decade, gave a memorial eulogistic of Roswell Park, student, scientist, surgeon, teacher, author and gentleman. At the conclusion thereof he read a resolution formulated by Drs. George Emerson Brewer, '84, Chairman, John Parmenter, '83, and E. R. McGuire, '01. On motion of Dr. DeLancey Rochester the resolution and memorial were unanimously adopted. On behalf of the graduating class (1914) President James M. Flynn presented a picture of Dr. Park to the University, containing the following inscription:

**ROSWELL PARK, A. M., M. D., LL. D., Professor of Surgery, 1883-1914. Died, February 15, 1914. Age 61 years. Presented by the Class of 1914. "His last class."**

Dean Herbert U. Williams accepted this picture on behalf of the Faculty.

### FREDERICK CARL BUSCH

A touching memorial on Dr. Busch was given by Professor A. T. Kerr, '97, of Ithaca, a life long friend, a fellow student in public school, high school, college, and this University, and his most intimate acquaintance. Dr. Kerr spoke very feelingly and recounted their joint experiences in education and study, the accomplishments of Dr. Busch's teaching in our University, in the Alumni Association, in his scientific investigations, in practical research and the infinite possibilities of a career cut short by an untimely death. The committee on memorial resolutions consisting of Henry J. Mulford, '89, Chairman; A. T. Kerr, '97, and Frederick J. Parmenter, '03, made its report through its chairman, and on motion of Dr. John R. Gray, '89, the memorial and resolution were both unanimously adopted. Dr. Henry J. Mulford then presented to the University with appropriate remarks a picture of Dr. Busch, bearing the following inscription:

**FREDERICK CARL BUSCH, B. S., M. D., '97. Professor of Physiology, 1900-1912. Died, January 3, 1914. Age, 40 years. Presented by a few of his many friends.**

On behalf of the Faculty Dean Williams accepted this picture.

### CLASS REUNIONS

The Class Reunions took place on Wednesday evening, June 3d. The class of 1874 in charge of Dr. John A. Pettit held its dinner at the Hotel Statler. Five members were present including Dr. Edward N. Brush of the Sheppard and Enoch Pratt Hospital. The boys of '79 were entertained at the University Club by Drs. Benjamin F. Rogers and Charles A. Wall. Under the direction of our sterling Alumnus, Dr. De Lancey Rochester, the class of '84 (sixteen in number) dined at the Saturn Club. The class of '89 held its 25th reunion at the Hotel Lenox the committee Dr. Henry J. Mulford and Dr. A. W. Bayliss, assembled twenty members. It was decided to elect Dr. Mulford permanent secretary putting him in charge of all class affairs. The reunion of the class of '94 was held at the Hotel Statler, twenty-two being present. Furthering the good work of the class committee, Drs. H. C. Rooth and F. S. Hoffman; it was decided to make the class reunion an annual affair. Drs. Julien Riester and Francis O'Gorman assembled twenty members of '99 at the Hotel Statler.

For enthusiasm and numbers the banner dinner was held in the Dutch Grill of the Hotel Statler when thirty-two boys of 1904 reunited. This class appointed permanent officers with Dr. Julius Richter as secretary and agreed to meet every year so as to further the interests of our Alma Mater. Drs. Ed. Koenig and Sam Moore deserve commendation for the excellent manner in which they perfected their arrangements. The baby reunion class (1909) under the direction of Ben Schreiner and Frank Brundage assembled for the first time since leaving College at the Teck Cafe.

The large attendance was particularly noteworthy in the number of out-of-town men present. The marked expressions of loyalty and enthusiasm for Alma Mater is more and more markedly evidenced from year to year and proves the advisability of the Classes reuniting at five year intervals rather than every ten years as was the custom formerly.

### THE ANNUAL DINNER

The Annual Dinner of our association which was the social event of our meeting took place on Thursday evening, June 4, at the Hotel Statler. There were two hundred members present. Our guests of honor were Prof. Ludwig Pick, Rabbi Louis J. Kopald, Drs. John A. Fordyce, George E. Brewer, '84, and Lewis Gregory Cole, of New York, and Dr. Edward N. Brush, '74, Towson, Md. After a most excellent repast thoroughly enjoyed by all, the toast master of the evening, Dr. Herman G. Matzinger, '84, was introduced by the president of our Association, Dr. Grover W. Wende, '89. Dr. Matzinger acquitted himself in his usual happy and felicitous manner, introducing each speaker with appropriate remarks. In the absence of Dr. Charles G. Stockton, Dr. Allen A. Jones, '89, toasted The Faculty. Our chief guest of honor, Professor Ludwig Pick of Berlin, responded to the toast "Endemic Diseases Peculiar to Germany." Prof. Pick spoke very wittily in faultless English and showed his marked versatility, being fully equal to the occasion in discussing Lésé Majesté, beer drinking and other conditions which we in America are not at all conversant with as are the inhabitants of the German Empire. The principal address of the evening was given by Rabbi Louis J. Kopald who discussed the topic "If I Were a Doctor" in which he commented on the relation of medicine to the ministry, the manifold opportunities afforded the physician in modern life and the possibilities of the profession in advancing civic interests and civic polity. Our distinguished fellow Alumnus Dr. E. N. Brush, '74, for many years actively interested in the Association responded on behalf of The Reunion Classes emphasizing our obligations to Alma Mater. James M. Flynn, president of the graduating class spoke for his class mates to the toast "Just Passed" so wittingly put by the Toast Master. Our Alumnae to the number of twenty-five in attendance were represented on the toast list by Dr. Mary Blair Moody, '76, who was the first woman graduate from our institution. Dr. Moody deserves special mention for coming from Los Angeles, Cal., to attend the meeting.

Dr. Peter W. Van Peyma, '72, now a member of the Council of the University of Buffalo and whom we always delight to hear, whether in the class room or at the festive board spoke fittingly on "A Laborer Worthy of His Hire." The celerity with which he completed the First and Second stages, and passed on to the Third was remarkable. Dr. George Emerson Brewer, '84, First Vice-president, spoke briefly on the character of the distinguished men constituting the faculty in the early eighties.

In presenting on behalf of the Class of 1904, a picture of Dr. John Parmenter, '83, Dr. Kauffman called the attention of the Alumni to the many years of faithful and devoted service as secretary and member of the faculty from which he retired ten years ago. Dean Williams accepted this picture for the faculty. The subjoined inscription expresses the class sentiment:

**John Parmenter, M. D., '83.**
**Professor of Anatomy and Clinical**
**Surgery, 1890-1904, when he retired.**
**Presented by the Class of 1904**
**In appreciation of his ability as a**
**teacher and his courtesy as a gentleman.**

Professor Park's picture hanging in the Roswell Park Memorial Room of the College Library, and Drs. Parmenter's and Busch's adorning the walls of the Library proper, will recall to our minds as we assemble from year to year the accomplishments of these men and will undoubtedly be a stimulus to the future students who come to pursue their medical studies in our Alma Mater. Let us hope that others of our illustrious teachers both gone or retired will be remembered by some of the classes at future reunions

The Banquet hall was tastily decorated with special banners for which credit is due Drs. Julius Richter and Edith R. Hatch. These banners are in the college colors, royal blue and white with the U. of B. diamond in the middle and the serpent entwined about a staff the insignium of medicine. These banners were arranged so as to alternate with Old Glory and in compliment to Prof. Pick a large German flag was used. Suspended from the ceiling were streamers of blue and white.

The singing in charge of Dr. Norman L. Burnham is deserving of special mention. The spirit and vim with which the songs were rendered continuously throughout the evening left no dull moment. Every speaker had a special song in his honor and besides our regular college songs, many of the popular songs of the day were rendered.

Dr. Kauffman, chairman of the Executive Committee announced the offering of a $10.00 prize to be given to that Alumnus of any department of the U. of B. who submits the best song before January 1, 1915, this song to be selected by a prize committee of impartial judges and rendered at the Annual University Day Dinner of the Federation of the Alumni Associations to be held in Buffalo on the evening of University Day (February 22, 1915). Let all our bards and song-writers set their muses aworking to the end that ultimately the U. of B. song book will be comparable to that of any other University in this land.

### FRATERNITY REUNIONS

The fraternity reunions were held on Tuesday night. The Nu Sigma Nu (I. C. I. chapter) and Phi Rho Sigma (Alpha Omega Delta chapter) were entertained at their respective chapter houses. The Omega Upsilon Phi Frat held its dinner at the Touraine Hotel.

### THE UNIVERSITY BISON

The attention of the Alumni is directed to the U. of B. paper **The University Bison** which is issued monthly and is edited and controlled by the student body. It behooves every loyal Alumnus to support this paper, the subscription price is only 25 cents per annum.

### REPRESENTATIVE ON THE UNIVERSITY COUNCIL

At the last Annual Meeting, Dr. Peter Van Peyma was elected representative of this association on the Council of the University for a period of four years to hold office as soon as a vacancy occurred. Dr. Van Peyma's appointment was approved by the council and he is now a member of the same. The import of this innovation cannot be too strongly emphasized as the Alumni will henceforth have a voice in the direction and managment of the University affairs, this being in line with all the up to date Universities.

### THE ALUMNI ASSOCIATION AND THE N. Y. STATE MEDICAL SOCIETY

In appreciation of his efforts to elevate the standard of medicine in this state, our retiring president. Dr. G. W. Wende, '89, was, last April, elected President of The Medical Society of the State of New York which is to meet in Buffalo on April 27, 28, and 29, 1915. A goodly attendance of our Alumni is expected and plans are under way for a dinner of our association on one of the evenings of the meeting of which due notice will be sent to the near-by Alumni at the proper time. It is a significant fact that out of the sixteen men who constitute the council of the State Medical Society, six are graduates of the University of Buffalo: President Grover W. Wende, '89; second vice-president, Dr. S. W. Toms, '90, Nyack, N. Y.; third vice-president, Dr. Robert M. Elliot, '90, Supt. Willard State Hospital, Willard, N. Y.; Dr. Thos. H. McKee, '98, Buffalo, chairman committee on scientific work; Dr. Albert T. Lytle, '93, Buffalo, chairman committee on arrangements and Dr. Arthur G. Bennett, '90, Buffalo, president eighth district branch of the state society.

The committee on scientific work in addition to Dr. McKee as chairman contains two U. of B. graduates, Dr. Franklin W. Barrows, '93, and Dr. James E. King, '95. Besides Dr. Lytle the committee on arrangements consists of Drs. A. L. Benedict, '88; Arthur G. Bennett, '90; Lesser Kauffman, '04; Edith R. Hatch, '06; Julius Richter, '04, and Edward Sharp, '98, all of Buffalo. The section officers are principally U. of B. graduates: Dr. James E. King, '95, chairman, section on Obstetrics: Dr. Nelson G. Russell, '95, section on medicine; Harry R. Trick, '01, secretary, section on surgery; Dr. DeWitt H. Sherman, '91, secretary, section on pediatrics and Dr. Lesser Kauffman, secretary of the newly formed section on syphilis.

### COMPARATIVE STATEMENTS OF ACTIVITIES FOR THE PAST THREE YEARS

| | 1912 | 1913 | 1914 |
|---|---|---|---|
| Registration .................... | 263 | 324 | 410 |
| Attendance at Annual Dinner ... | 130 | 175 | 200 |
| Balance in Treasury ........... | $132 | $260 | $250 |

This statement shows a steady and healthy increase in the activities of our Association from year to year and were it not for the increased expenses of this year our efficient treasurer, Dr. W. F. Jacobs, would have had a balance of $400.00.

### OUR PLANS FOR THE COMING YEAR

Encouraged by this most excellent showing, the Executive Committee in planning its arrangements for the ensuing year asks your favorable consideration and hearty support for the following:

1—ALUMNI CATALOGUE to be published this year requires data from many of our Alumni to make it complete. Will you therefore co-operate with us in filling out cards sent to you later?

2—LOCAL ALUMNI MEETINGS to be held by The Interstate Alumni Association, The Rochester District Association and the new associations to be formed this year.

3—ANNUAL DINNER of the Federated Alumni Association (all departmnts of the U. of B.) on University Day (February 22, 1915).

4—ALUMNI DINNER in April, 1915, during the N. Y. State Medical Meeting.

5—FORTIETH ANNUAL MEETING—Tuesday to Friday, June 1st to 4th, 1915, with all its distinctive features. Arrangements are already under way to make this even more successful than its predecessors. Two men of national prominence have accepted invitations for clinics. While the officers are at all times ready and willing to do their part it is essential that the entire Alumni body give them their most earnest cooperation and support. We look forward to a registration of at least 600.

**Reunions** will be held by the classes of '65, '70, '75, '80, '85, '90, '95, '00, '05, and '10, the same to be in charge of the respective class committees. This year the class of 1904 has set a pace; thirty-two members at class dinner, twenty at Alumni Dinner, presentation of picture of Dr. Parmenter to the University. It therefore behooves the **naughty fives** to get busy early and equal this enthusiastic class. Why not go them some better? Whether a member of the reunion classes or not, all Alumni are urged to be present at our fortieth anniversary meeting and show their enthusiasm for and loyalty to their Alma Mater.

Arrangements have been made with Dr. A. L. Benedict, '88, editor of the BUFFALO MEDICAL JOURNAL in which this report is published to have an Alumni page wherein all announcements of the association will appear and any news item sent to the editor will be published therein.

### Statement Made by Dean Williams of the Medical Department at the Business Meeting of the Alumni Association.

It is with pleasure that I report to the Alumni once more that the Medical Department has had a successful year. The number of students in the college is large, about 230, and they are a promising lot of young people, and seem to be doing good work. The entering class is unusually large, being over 90. As the Alumni are aware, the entrance requirements of the Medical Department have been raised, so that beginning with September, 1914, entering students will be obliged to have a year of college work in Physics, Chemistry, Biology and a modern language, in addition to the regular four years of high school work. To meet this necessity the University has been conducting courses in these subjects, which we call "Pre-Medical Courses." About twenty-seven students have been taking the pre-medical course during the present year. We shall necessarily have a considerably smaller freshman class in the Medical Department than we have had for some years past, but we expect that it will reach the former level after a few sessions.

It is pleasant to inform the Alumni that Dr. Irving M. Snow, Professor of Pediatrics, who made the Medical Department a gift of a thousand dollars a year ago, has added to this a second thousand dollars within a few weeks. The Faculty has decided to keep Dr. Snow's gift intact, and it will be known as "**The Irving M. Snow Fund.**"

I am glad to have this opportunity to call the attention of the Alumni to the University paper called "The Bison," which is issued monthly during the college year, and contains a great deal of matter that is of interest to our graduates. I hope they will give it their cordial support and become subscribers and send items of interest to the readers.

The college has, of course, suffered an irreparable loss in the death of Dr. Roswell Park, and we are all deeply grieved at the death of Dr. F. C. Busch, to whom we were all warmly attached, although he had recently resigned his teaching position. It is not necessary to speak on this subject fully at present as memorials to Dr. Park and Dr. Busch form an important part of this meeting. The Alumni will be interested to know, however, that the Faculty has decided that for the present it will not undertake to fill the Chair of Surgery formerly occupied by Dr. Park, whose associates and assistants have signified their willingness to conduct the work until some permanent arrangement can be made.

---

**The Oneida Co. Medical Society** met July 8. Wm. B. Reid of Rome was expelled for unethical advertising, the case having been delayed by injunction since 1909. Three other Rome physicians, associated with Dr. Reid, were considered not guilty of unethical advertising. Drs. Walter J. Smith of Waterville, Harry T. Remmer and James W. Byrne of Utica were admitted to membership. Dr. J. M. Wainwright of Scranton read a paper on the Role of the Caecum in Chronic Disorders of the Right Lower Abdomen. The Society accepted the invitation of the Hawthorne Farm of Clinton, to investigate the subject of Clean Milk by a visit to the farm July 25. Various papers were promised for this special meeting.

---

**The American College of Surgeons** met at the Bellevue-Stratford in Philadelphia, in June. There was an attendance of over 800. 1,100 new fellows were admitted, making a total of more than 3,200. In less than an hour, $100,000 was subscribed toward an endowment of $500,000. This is desired to secure a permanent home for the College and to maintain various bureaus, with paid assistants and expert surgeons, to conduct the business of the College itself, investigate undergraduate and post-graduate medical instruction, assist in standardizing hospitals and organize legislative and civic committees. It is estimated that the College will require at least $35,000 a year to carry on these activities, of which only a little more than $15,000 will be received from dues. Clinics and scientific papers were given for the members. The officers of the College are as follows:

President—Dr. J. M. T. Finney.

First Vice-president—Dr. W. W. Chipman.

Second Vice-president—Dr. Rudolph Matas.

General Secretary—Dr. Franklin H. Martin.

Treasurer—Dr. Albert J. Ochsner.

Board of Regents—Dr. George E. Armstrong, Dr. George E. Brewer, Dr. Herbert A. Bruce, Dr. Frederick J. Colton, Dr. George W. Crile, Dr. J. M. T. Finney, Dr. William Haggard, Dr. Edward Martin, Dr. Franklin H. Martin, Dr. Charles H. Mayo, Dr. Robert E. MacKechnie, Dr. John B. Murphy, Dr. Albert J. Ochsner, Dr. Harry M. Sherman and Dr. Charles F. Stokes.

We take this occasion to say that when the college was first proposed, it seemed advisable to give a somewhat guarded prognosis. This course was justified by statements made by its originators, in conjunction with past experience with somewhat similar undertakings. The unparallelled rapidity with which the preliminary plans have been matured and with which so large a membership of picked men has been enrolled, indicate that there has been a genuine demand for such an organization. The assurances of a broad minded policy both in the selection of fellows and in the activities of the organization by men of unquestioned standing and indubitable sincerity and the earnest endeavor to avoid any action that might be adversely criticized, ought to suffice to lay the various ghosts that some of our contemporaries have been seeing.

---

**The Gross Medical Club** held their meeting at Akron, N. Y., July 10th, as guests of Dr. F. A. Helwig. Dr. Chester C. Cott of Buffalo presented a paper on diseased conditions of the tonsils. A very lively discussion followed, especially by Dr. Albert J. Colton who brought out many points of interest regarding the tonsils in connection with rheumatic conditions. By special invitation the members and their wives visited the canning factory, where at the present time large quantities of peas are being prepared for the N. Y. market.

---

**The Medical Society of the State of New York** has established a Section on Syphilis; Chairman, John A. Fordyce of New York, Secretary, Lesser Kauffman of Buffalo. It is expected that five sessions will be held at the meeting in Buffalo in 1915.

